# Protective effect of berberine chloride against cisplatin-induced ototoxicity

**DOI:** 10.1007/s13258-021-01157-1

**Published:** 2021-11-20

**Authors:** Jong-Heun Kim, Jeong-In Baek, In-Kyu Lee, Un-Kyung Kim, Ye-Ri Kim, Kyu-Yup Lee

**Affiliations:** 1 Department of Biology, College of Natural Sciences, Kyungpook National University, Daegu, Republic of Korea; 2 School of Life Sciences, KNU Creative BioResearch Group (BK21 Plus Project), Kyungpook National University, Daegu, Republic of Korea; 3 Department of Aroma-Applied Industry, College of Herbal Bio-Industry, Daegu Haany University, Gyeongsan, 38610 Republic of Korea; 4 Department of Internal Medicine, Research Institute of Aging and Metabolism, School of Medicine, Kyungpook National University, Daegu, Republic of Korea; 5 Advanced Bio-Resource Research Center, Kyungpook National University, Daegu, Republic of Korea; 6 Department of Otorhinolaryngology-Head and Neck Surgery, School of Medicine, Kyungpook National University, Daegu, 41944 Republic of Korea

**Keywords:** Cisplatin, Ototoxicity, Berberine chloride, Antioxidant, ROS

## Abstract

**Background:**

Cisplatin (CP) is an effective anticancer drug broadly used for various types of cancers, but it has shown ototoxicity that results from oxidative stress. Berberine has been reported for its anti-oxidative stress suggesting its therapeutic potential for many diseases such as colitis, diabetes, and vascular dementia.

**Objective:**

Organ of Corti of postnatal day 3 mouse cochlear explants were used to compare hair cells after the treatment with cisplatin alone or with berberine chloride (BC) followed by CP.

**Methods:**

We investigated the potential of the anti-oxidative effect of BC against the cisplatin-induced ototoxicity. We observed a reduced aberrant bundle of stereocilia in hair cells in CP with BC pre-treated group. Caspase-3 immunofluorescence and TUNEL assay supported the hypothesis that BC attenuates the apoptotic signals induced by CP. Reactive oxygen species level in the mitochondria were investigated by MitoSOX Red staining and the mitochondrial membrane potentials were compared by JC-1 assay.

**Results:**

BC decreased ROS generation with preserved mitochondrial membrane potentials in mitochondria as well as reduced DNA fragmentation in hair cells. In summary, our data indicate that BC might act as antioxidant against CP by reducing the stress in mitochondria resulting in cell survival.

**Conclusion:**

Our result suggests the therapeutic potential of BC for prevention of the detrimental effect of CP-induced ototoxicity.

## Introduction

Hearing loss is a symptom of having an impaired perception of various tunes and sounds. According to the WHO, 460 million people are suffering from hearing loss, and the number is expected to reach 900 million by 2050 (Olusanya et al. 2019). Hearing loss can be classified into congenital and acquired loss of hearing. Congenital loss of hearing from birth is due to several reasons such as premature birth, genetic factors, and congenital infections (Korver et al. 2017). In contrast, normal hearing at birth can be impaired by multiple causes including exposure to noise, aging, and ototoxic drugs (Joo et al. 2018).

Ototoxicity refers to a drug-induced side effect that causes damage to auditory or vestibular tissue cells which prevents them from functioning normally. There are over 600 categories of drugs known to cause ototoxic hearing loss. Amino-glycoside antibiotics, platinum-based chemotherapy, loop diuretics, macrolide antibiotics, antimalarial drugs, etc. are highly effective in treating infections and tumors in both children and adults (Ganesan et al. 2018). In particular, cisplatin is a widely used anticancer drug for solid cancer in ovaries, lung, bladder, and testis and has been used since the 1970s, and ototoxicity is known as a representative side effect (Boulikas and Vougiouka 2004). Between 40 and 80% of patients who took cisplatin experienced permanent hearing impairment, as well as tinnitus, dizziness, and ear pain (Rybak et al. 2009; Santos et al. 2020). Due to the anatomical structure of the cochlea which is isolated as well as almost a closed-system, cisplatin cannot be released as fast as the rate of its accumulation (Rybak et al. 2009). This eventually leads to cellular damage and apoptosis in the cochlear tissue followed by hearing loss.

The causes of cisplatin damage have been known to be associated with excessive reactive oxygen species (ROS). In in vivo and in vitro experiments, cisplatin accumulation in inner hair cells (IHCs), outer hair cells (OHCs), stria vascularis, and spiral ganglion neurons exhibited abnormally high concentrations of ROS and a reduction of the antioxidant enzymes by binding with cochlear tissue DNA (Santos et al. 2020). ROS generation induced by cisplatin may provoke tissue damage causing increased of apoptosis which consequently accelerates ROS generation repeatedly (Gentilin et al. 2019; Rybak et al. 2009; Rybak and Whitworth 2005). Therefore, to reduce the cisplatin-induced apoptosis, drugs that can reduce the excessive ROS generation may be required.

Berberine is an alkaline isoquinoline commonly found in the genus Berberis within the Berberidaceae family (Neag et al. 2018). Because of its insolubility, salt forms such as berberine chloride and berberine sulfate are commonly available in the form of tablets or capsules (Battu et al. 2010). Various therapeutic outcomes such as anti-inflammation, anti-oxidative stress, and neuroprotective effects have been reported (Aski et al. 2018; Yin et al. 2008; Zhou and Mineshita 2000). For instance, Zhou et al. reported that berberine reduced colonic mucosal colitis in rats, and Aski et al. observed the neuroprotective effect of berberine in chronic cerebral hypoperfusion suggesting a therapeutic potential for diseases such as colitis, and vascular dementia (Zhou and Mineshita 2000; Aski et al. 2018). Furthermore, numerous researchers have shown that the therapeutic challenge of berberine treatment to ameliorate cisplatin-induced toxicity exhibited reasonable efficacy in various organs including pancreas, kidney, and lung (Chandirasegaran et al. 2017; Domitrovic et al. 2013; Mo et al. 2014).

However, the protective effect of berberine chloride for cisplatin-induced ototoxicity has not yet been identified. Hence, we investigated the potential of berberine chloride as a protective agent against cisplatin-induced ototoxicity in murine cochlear explants by focusing on ROS generation and apoptosis of hair cells.

## Materials and methods

### Culture of mouse cochlear explants

Primary cochlear explants were extracted from postnatal day 3 Institute for Cancer Research mice. The dissected organs of Corti were attached to the culture dishes and then incubated with high-glucose Dulbecco’s Modified Eagle’s Medium (DMEM; Hyclone, USA) containing 10% fetal bovine serum (FBS; Hyclone, USA) and ampicillin (10 μg/mL; Life Technologies, USA) in humidified atmosphere of 5% CO_2_ at 37 °C.

### Histological evaluation

To determine the protective effects of berberine chloride against cisplatin-induced ototoxicity in the organ of Corti, we investigated morphological defects of IHCs and OHCs by staining stereocilia with phalloidin. At the end of the 30 h incubation period, all cochlear explants were washed with phosphate buffered saline (PBS), and fixed with 4% paraformaldehyde in PBS. Permeabilization and staining was conducted simultaneously by Alexa Flour488 conjugated phalloidin (Invitrogen-Molecular Probes, USA) in 0.1% Triton X-100 in PBS for 1 h at room temperature (RT).

### Analysis of apoptotic cell death

Immunohistochemistry for caspase-3 and terminal deoxynucleotidyl transferase dUTP nick end labeling (TUNEL) assay were carried out to determine if berberine chloride protects cells from cisplatin-induced apoptosis. At the end of the 30 h incubation periods, samples were permeabilized with 0.1% PBS-Tx and blocked with 5% normal goat serum. Anti-active caspase-3 (Cell Signaling Technology, USA) was treated and then stained with Alexa Fluor 488 or 555-conjugated phalloidin (Invitrogen-Molecular Probes, USA).

DNA fragmentation was evaluated by TUNEL assay according to the manufacturer’s protocol (Promega, USA). The samples were permeabilized with 0.1% Triton X-100 and 0.1% sodium citrate in distilled water and stained with TUNEL working solution.

### Detection of mitochondrial ROS

MitoSOX-red (Invitrogen, USA) were used as a mitochondria-specific fluorogenic indicator of superoxide. At the end of the 30 h incubation periods, all cochlear explants were washed with PBS and stained with MitoSOX-red (5 μM) for 10 min in a humidified atmosphere of 5% CO_2_ at 37 °C. After washing with PBS, the specimens were visualized using a Zeiss Axio Imager A2 fluorescence microscope.

### Determination of mitochondrial membrane potential (MMP, ΔΨm)

MMP was determined by the MitoProbe™ JC-1 (Molecular Probes, Eugene, OR, USA) according to the manufacturer’s instructions. At the end of the 30 h incubation periods, all cochlear explants were washed with PBS and incubated with JC-1 (2 µM) for 50 min in 5% CO_2_ at 37 °C in the dark. To examine the sensitivity of JC-1, 50 mM of carbonyl cyanide 3-chlorophenylhydrazone (CCCP) were added to the cochlear explants that were not treated with any drugs before treatment of JC-1 and incubated for 5 min at 37 °C. We quantified red over green JC-1 fluorescence ratios by splitting the images according to color channels and measured the average grayscale using Image J software (http://imagej.nih.gov/ij/).

## Results and discussion

In this study, we investigated whether BC is efficient in preventing CP caused ototoxicity in postnatal day 3 mouse cochlear explants. Berberine has been reported for its potential use in various conditions such as anticancer, anti-oxidative, anti-inflammatory, infection, diabetes and many others (Cernakova and Kostalova 2002; Li et al. 2014; Neag et al. 2018; Sun et al. 2009; Yin et al. 2008). Research has been carried out suggesting the therapeutic possibility of berberine in various organs, and yet studies of BC efficacy on CP ototoxicity have not been conducted. Hence, we focused on the capacity of BC in suppressing CP induced toxicity in the organ of Corti.

**Fig. 1:**
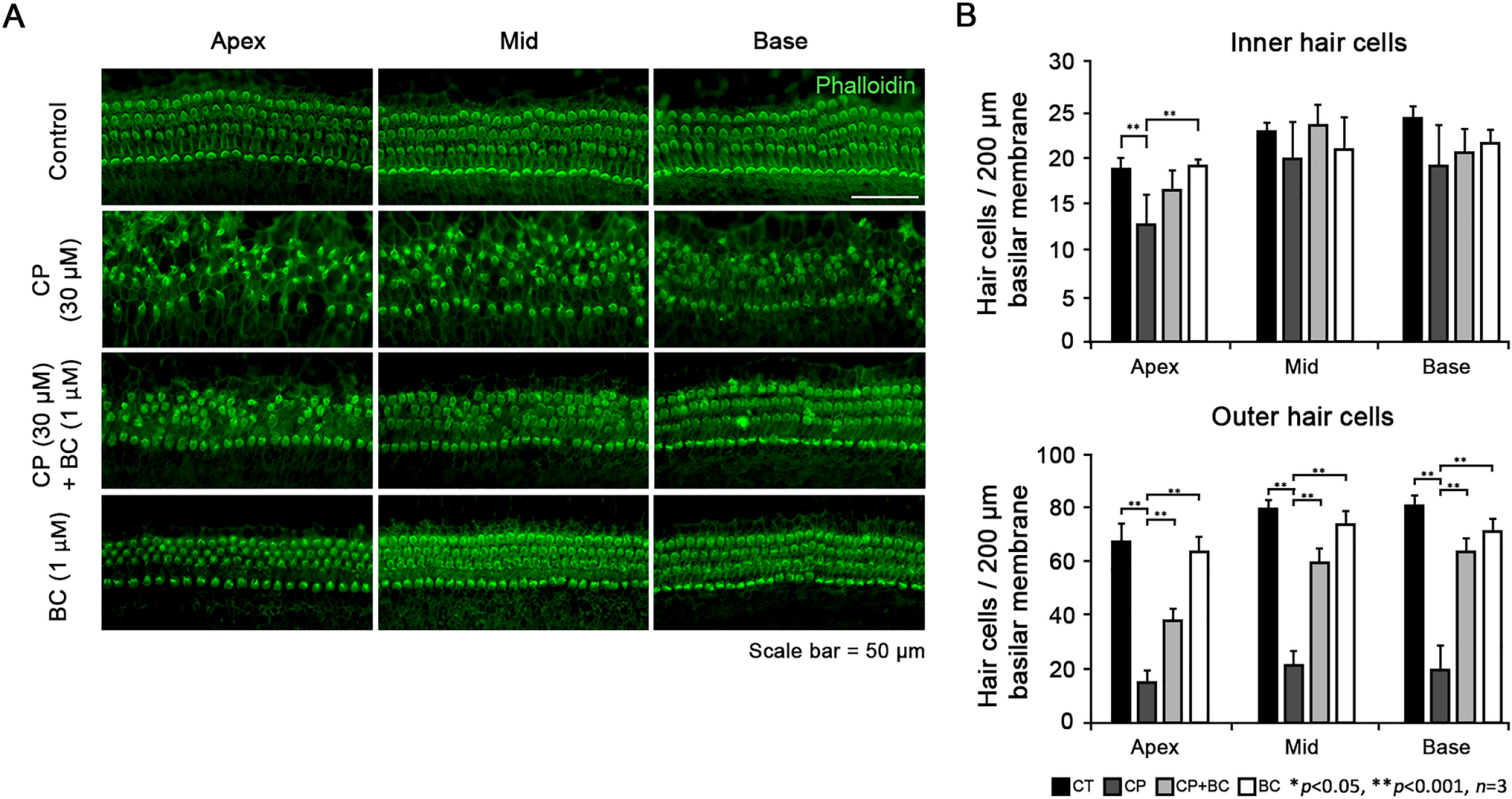
Effect of berberine chloride on cisplatin-induced ototoxicity. **A** Images of immunofluorescence representing the organ of Corti explants of four experimental groups (CT, CP alone, CP + BC, and BC alone). Stereocilia were stained with phalloidin (green). **B** The y-axis indicates the average number of normal stereocilia of IHCs or OHCs in the organ of Corti in the 200 μm region of three turns—apical, middle, and base. Data are presented as mean ± SD. Scale bar: 50 μm. n = 3 for each group, *p < 0.05 and **p < 0.001

First of all, we conducted phalloidin staining to determine whether BC is actually effective for hair cells to survive CP treatment. Figure 1A shows the fluorescence results of phalloidin staining of the cochlear explants in three parts divided by apex, mid, and base for each experimental group. In the control (CT) group, without any BC or CP treatment, postnatal day 3 murine organ of Corti exhibited undamaged three rows of OHCs and a row of IHCs respectively. In contrast, in the organ of Corti of the 30 μM of CP treated group, we observed evident disruption in morphology of the stereocilia and rows of hair cells while OHCs are found to be more damaged then IHCs. CP + BC group, when 1 μM of BC was pretreated an hour prior to CP, both IHCs and OHCs were almost restored to the natural 3 + 1 rows. The result of the BC group was similar to that of the CT group.

To estimate the protective effect of BC statistically, surviving hair cells in apex, mid, and base of the IHCs and OHCs were counted respectively (n = 3 for each group, *p < 0.05 and **p < 0.001, Fig. 1B). In the IHCs, only apex exhibited a significantly reduced number of surviving hair cells by CP treatment while the other portions including mid and base were not severely affected. Even though the number of surviving IHCs reduced by CP in all portions tends to be recovered by the treatment of BC, none of them indicated statistical significance. On the contrary, number of surviving OHCs was decreased significantly by CP treatment in all portions. However, in OHCs, CP with BC pre-treated group (CP + BC group) in all portions showed a significantly increased level of cell survival. In addition, no significant detrimental effects of BC were found as there were no statistical differences in hair cell counts of the BC group compared to the CT group in both of IHCs and OHCs (statistic data not shown).

Interestingly, we could observe that IHCs had a more distinct morphology of stereocilia with an intact row compared to OHCs throughout the whole phalloidin stained experimental groups. It has been reported that in organ of Corti, susceptibility to cisplatin differs as a row of IHCs are more resistant than three rows of OHCs (Schacht et al. 2012). As shown in Fig. 1B, our data exhibited consistently that the OHCs were more vulnerable to CP compared to IHCs which may be because cisplatin uptake in three rows of OHCs occurs prior to IHC uptake of cisplatin in the organ of Corti (Ding et al. 2012).

**Fig. 2:**
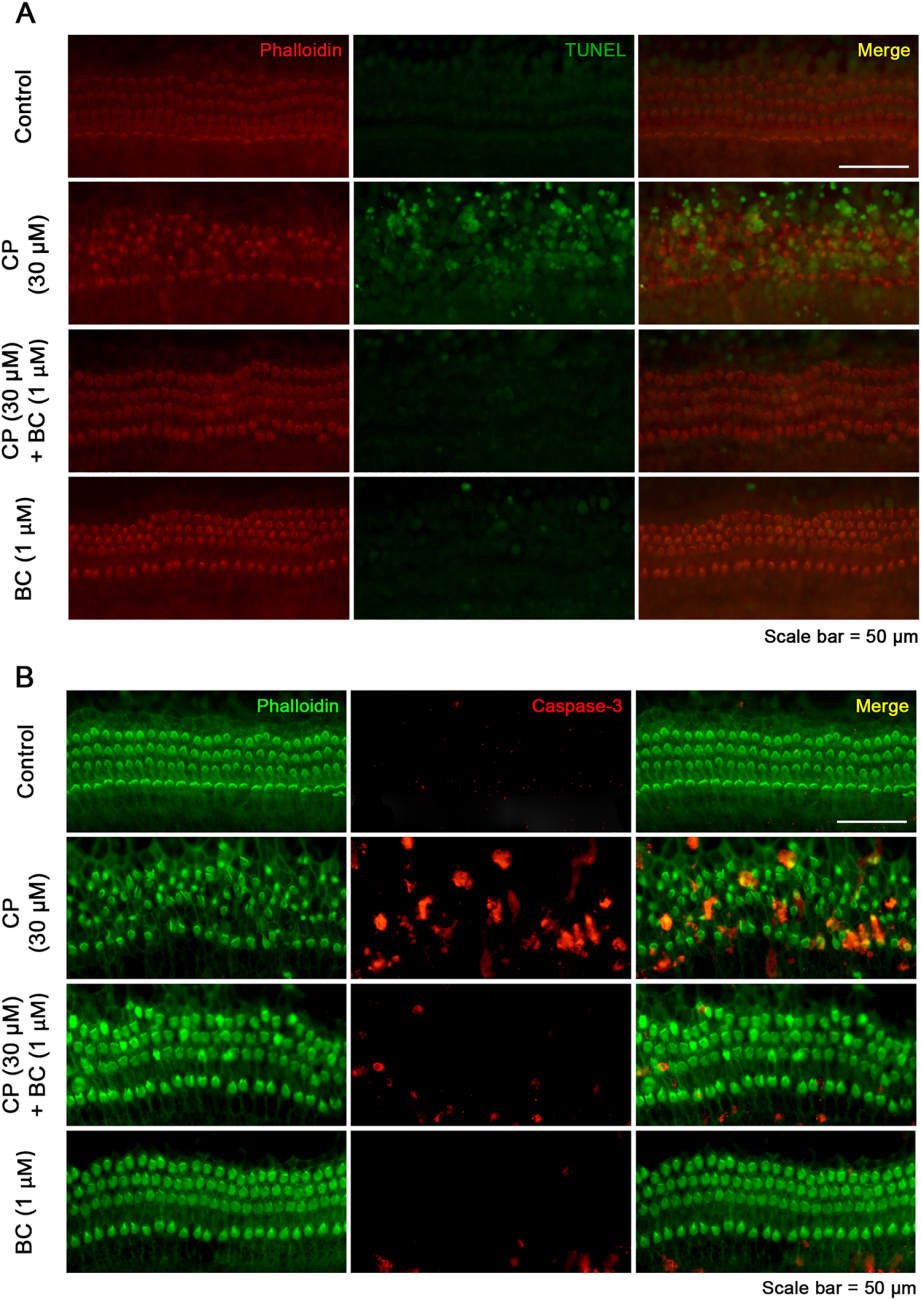
Effects of cisplatin and berberine chloride on DNA damage and apoptosis in the organ of Corti. Active caspase-3 immunofluorescence and TUNEL assay were used to determine DNA damage and apoptotic cells in cochlear explants. **A** Reduced DNA damage by BC treatment was observed by labeling phalloidin in red, TUNEL signal in green. **B** Cells were labeled to represent active caspase-3 in red, phalloidin in green to compare the apoptotic signal in presence of BC. Scale bar: 50 μm

After examining whether BC could ameliorate the cisplatin toxicity and enhance cell survival by phalloidin staining, we assessed the fluorescence intensity of apoptotic signals of hair cells in each experimental groups by detecting damaged DNA using TUNEL assay, and by detecting the activated cleaved caspase-3 to confirm the efficacy of BC (n = 3 for each group, Fig. 2). In Fig. 2A, stereocilia of the hair cells in the organ of Corti emitted red fluorescence in all experimental groups consistent with the results of Fig. 1A. Also, we observed high levels of green fluorescence signals bound to damaged, nicked DNA by using the TUNEL assay in hair cells of the CP group, while only faint TUNEL signals were detected in both of the CT and BC group. Meanwhile, the result of the CP + BC group exhibited a dramatically decreased level of the TUNEL signal which could barely be detected in cochlear explants. Figure 2B mainly describes the activation of the apoptotic signal in CP and BC related conditions. Stereocilia of the hair cells were stained green with phalloidin, which were consistent with the results of Figs. 1A, and 2A. On the other hand, the red immunofluorescence indicating cleaved caspase-3, which is an activated form of caspase-3, were absent in the cochlear explants in both of the CT and BC groups while the red fluorescence appeared to be all over the cochlear explants when CP were treated alone. The intensity of the red apoptotic fluorescence signal in CP + BC group reduced substantially, resulting in similar results to those of the TUNEL assay demonstrating apoptosis preventing the efficacy of BC against CP.

We could find fragmentation of DNA using the TUNEL assay followed by active cleaved caspase-3 immunofluorescence signals confirming that one of the main factors for apoptosis might be excessive ROS generation by CP. We detected cleaved, activated caspase-3 to assess cellular apoptosis by immunofluorescence because caspase is a downstream executioner protein which activates apoptosis related substrates including DNA repair enzymes, DNA fragment factor, and cytoskeletal proteins.

**Fig. 3:**
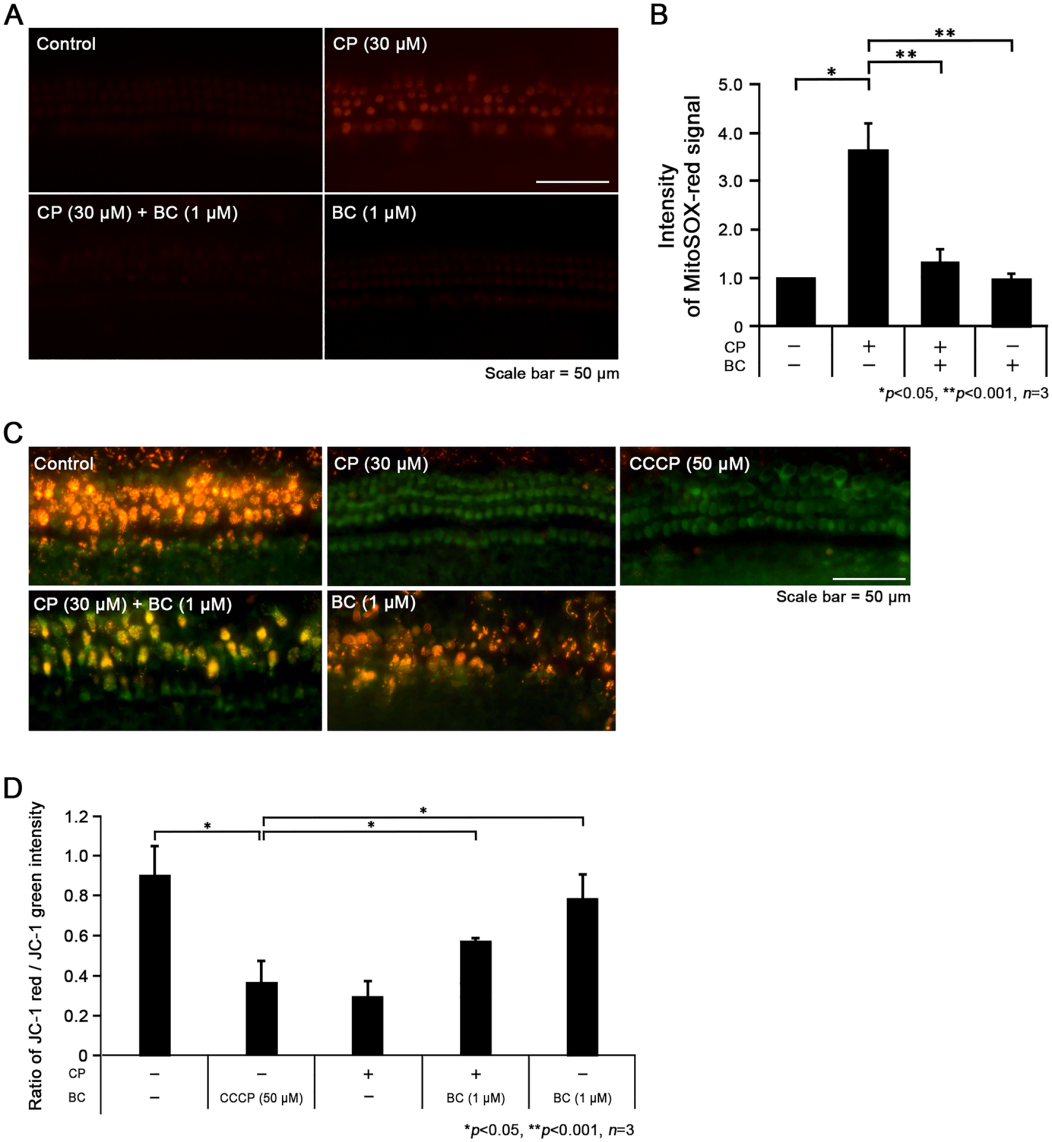
Protective effect of berberine chloride on mitochondrial oxidative stress caused by cisplatin. **A** Generation of mitochondrial ROS in cochlear explants observed by MitoSOX-red staining. **B** Statistical data showed a decreased number of MitoSOX-red signals when berberine chloride was pretreated before cisplatin. **C** Fluorescence of JC-1 assay representing the state of mitochondrial membrane potential. Immunofluorescence signal images of the organ of Corti explants of the various experimental groups. **D** Mitochondrial depolarization is assessed by comparing the red/green fluorescence intensity ratio. Data are presented as mean ± SD. Scale bar: 50 μm. n = 3 for each group, *p < 0.05 and **p < 0.001

We confirmed that CP leads to hair cell apoptosis in the murine cochlear explants while pretreatment of BC could prevent the ototoxicity by applying phalloidin staining, TUNEL assay, and immunofluorescence. Next, we focused on the effect of CP and BC in regard to mitochondrial ROS generation because the main reason of CP ototoxicity is well known for generation of highly reactive free radicals from mitochondrial ROS. We used MitoSOX-red to detect superoxide production in the mitochondria and also MitoProbe™ JC-1 to depict the mitochondrial membrane potential (MMP) in cochlear explants (Fig. 3).

The MitoSOX-red signal appeared significantly in the cochlear explants and extensively in the CP group indicating the production of excessive ROS. However, not only the CT or BC group but also CP + BC group exhibited only a faint intensity of the MitoSOX-red signal demonstrating the reduced generation of mitochondrial ROS (Fig. 3A). Image J was applied to compare the intensity of MitoSOX-red fluorescence between all experimental groups (n = 3 for each group, *p < 0.05 and **p < 0.001; Fig. 3B). The data displayed that the intensity level of the CP group exceeded an almost three times higher intensity than that of the CT or BC groups. The CP + BC group showed a dramatic decrease of almost 1/3 in the level of MitoSOX-red intensity compared to the CP group implying the efficacy of BC in reducing mitochondrial ROS generation.

Mechanisms regarding the efficacy of berberine protection of cells from ROS vary in many anti-oxidative related research. One of the main target of berberine is the AMPK pathway, which activates the Nrf2 pathway upregulating the expression of anti-oxidative genes including HO-1, NQO-1, GST as well as increasing the level of antioxidant enzymes such as GSH, GSH-Px, and SOD (Li et al. 2014; Mo et al. 2014). Even though we did not assess the expression of these antioxidant factors directly, we could assume that at least one of these antioxidant systems were reinforced by BC because the level of ROS generation in the presence of CP significantly reduced when BC were pretreated in the cochlear explants. Furthermore, the result of reduced MMP disruption in CP + BC group also supports the anti-oxidative reinforcement function of BC.

Subsequently, we assessed the MMP by using the MitoProbe™ JC-1 assay to verify whether the protective effect of BC, which reduced ROS generation, actually preserves MMP in spite of the CP treatment (Fig. 3C). CCCP is an agent used to inhibit MMP generation so that the green to red fluorescence shift of the JC-1 probe does not occur. Both of the CCCP and CP groups showed general green fluorescence, indicating that MMP were disrupted by CP treatment. We observed red fluorescence overlapped with green fluorescence in both of the CT and BC groups. In addition, CP + BC group displayed overlap of the red and green fluorescence even in presence of CP which indicated that the MMP were preserved by the protective efficacy of BC. The ratio of JC-1 red/green significantly decreased in both the CCCP and CP groups when compared to the ratio of the CT group indicating the malfunction of MMP generation (n = 3 for each group, *p < 0.05 and **p < 0.001; Fig. 3D). On the contrary, unlike the CP group, the CP + BC group partially restored the JC-1 red/green ratio significantly indicating that BC could be helpful in preserving MMP from the deleterious effect of CP.

Our study was the first to use BC to reduce the ototoxicity of CP. An interesting observation from these experiments was that the protective effect of BC in OHC was shown not only in the mid and base areas but also in the apex. Other ex vivo experiments were conducted using drugs such as KL1333 and KPR-A020 to ameliorate the ototoxicity of CP. In contrast to KL1333 which showed cellular protection in mid and base only, BC treatment expressed higher efficacy of OHC survival not only in the mid and base region but also in the apex as well. In the case of KPR-A020, the cell protection effect was similar to that of BC throughout all regions but the concentration of CP treatment was 20 μM which was lower than the concentration of CP used in this experiment (Kim et al. 2017; Lee et al. 2020).

The exact mechanisms for how BC protects the whole region of the cochlea from CP ototoxicity have not yet been elucidated. Even though we confirmed that BC reduces mitochondrial ROS stress in cochlea, a specific explanation for how BC reduces mitochondrial ROS stress is unknown. Subsequent studies may elucidate the specific protective effect of BC in cochlear hair cells. In summary, our study suggests the therapeutic potential of BC against CP induced ototoxicity through reducing the mitochondrial ROS stress leading to hair cell survival.
